# Alcohol Consumption and Cardiovascular Disease: A Narrative Review of Evolving Perspectives and Long-Term Implications

**DOI:** 10.3390/life14091134

**Published:** 2024-09-09

**Authors:** Ovidiu Stefan Georgescu, Liviu Martin, Georgică Costinel Târtea, Alexandra-Daniela Rotaru-Zavaleanu, Sorin Nicolae Dinescu, Ramona Constantina Vasile, Andrei Gresita, Veronica Gheorman, Madalina Aldea, Venera Cristina Dinescu

**Affiliations:** 1 Doctoral School, University of Medicine and Pharmacy of Craiova, 2 Petru Rares Str., 200349 Craiova, Romania; georgescuovi@yahoo.com; 2 Faculty of Medical Care, Titu Maiorescu University, Văcărești Road, no 187, 040051 Bucharest, Romania; liviu.martin@prof.utm.ro; 3 Department of Physiology, University of Medicine and Pharmacy of Craiova, 2 Petru Rares Str., 200349 Craiova, Romania; georgetartea@gmail.com; 4 Department of Epidemiology, University of Medicine and Pharmacy of Craiova, 2 Petru Rares Str., 200349 Craiova, Romania; alexandra.rotaru@umfcv.ro (A.-D.R.-Z.); sorin.dinescu@umfcv.ro (S.N.D.); 5 Department 3 Medical Semiology, University of Medicine and Pharmacy of Craiova, 2 Petru Rares Str., 200349 Craiova, Romania; veronica.gheorman@umfcv.ro; 6 Department of Psychiatry, University of Medicine and Pharmacy of Craiova, 2 Petru Rares Str., 200349 Craiova, Romania; madalina.aldea@umfcv.ro; 7 Department of Health Promotion and Occupational Medicine, University of Medicine and Pharmacy of Craiova, 2 Petru Rares Str., 200349 Craiova, Romania; veneradi@yahoo.com

**Keywords:** alcohol consumption, cardiovascular disease, heavy drinking, binge drinking, moderate drinking, hypertension, myocardial ischemia

## Abstract

Cardiovascular illnesses remain the primary cause of death, accounting for at least 17.9 million fatalities per year and posing a significant public health problem because of its extensive predominance and effect on healthcare systems. The etiology of cardiovascular disease is complex and involves several environmental and lifestyle factors. Alcohol use is a highly important determinant because of its dual-edged effect on cardiovascular health. Multiple studies indicate that moderate alcohol consumption may have certain advantages, such as slight enhancements in lipid profiles. Conversely, excessive alcohol intake is associated with serious negative consequences, including cardiomyopathy, hypertension, arrhythmias, and even mortality. The aim of this study is to provide a comprehensive analysis of the several effects of alcohol on cardiovascular health and their understanding within the medical field over time. It uses an interpretative narrative review methodology and analyzes studies that focus on genetic risk factors, gender differences, and shifts in paradigms in recent years. This article highlights the need for obtaining a thorough understanding of the effects of alcohol on cardiovascular health to support public health guidelines and clinical practice, and it underscores the significance of including alcohol consumption into the broader context of cardiovascular risk management and identifies important subjects for further study.

## 1. Introduction

One of the most frequent causes of mortality worldwide continues to be represented by cardiovascular diseases (CVD), which contribute to approximately 17.9 million deaths annually while posing a considerable public health challenge due to their increased prevalence and the substantial strain they place on global healthcare systems [1]. The causes of cardiovascular disease are complex, with many environmental and lifestyle determinants that complicate both preventive and therapeutic approaches [2]. Dietary patterns, including excessive consumption of saturated fats, trans fats, and salt, greatly elevate the likelihood of cardiovascular disease while poor engagement in physical exercise worsens this risk by causing obesity, insulin resistance, and the development of type 2 diabetes [3,4]. Air pollution, as well as toxic chemical exposure, can also be a cause of inflammation that can lead to cardiovascular damage [5]. Workplace dangers such as silica dust or hazardous chemicals can also deteriorate heart function [6,7].

Excessive alcohol consumption and cigarette smoke toxins have a profound detrimental effect on cardiovascular health [8]. Although modest levels of alcohol consumption may provide certain advantages, such as enhanced lipid profiles, excessive alcohol consumption presents significant hazards [9]. The condition is associated with liver illness, cardiovascular diseases, neurological pathologies, cancer, and mental health difficulties [10]. The dangers may vary according to age, with young individuals encountering dangerous conduct and accidents, while older persons bear the burden of chronic illnesses and accelerated aging caused by reduced alcohol metabolism [11,12]. Alcohol can lead to the appearance of arrhythmias, cardiomyopathy, and even hypertension [13]. It is essential to fully understand the age-related variability of alcohol use to establish the most effective treatment approaches. Older patients are the most vulnerable when it comes to cardiovascular disorders, and their treatments must be cautiously adjusted, especially in patients who have a higher alcohol intake. Furthermore, it is crucial to clarify the dose–response relationship and fundamental mechanisms of alcohol’s effect on cardiovascular health to establish evidence-based public health guidelines and focused interventions.

Cardiovascular disease requires a comprehensive examination of all modifiable risk factors, including lifestyle behavior like alcohol consumption [14]. These efforts are crucial for mitigating cardiovascular risk and improving patient outcomes. Alcohol use has a notable impact on CVD, and it influences both the preventive and the therapeutic approaches [13,15]. This review provides an examination of the many impacts that alcohol has on cardiovascular health, emphasizing both its conceivable advantages and notable hazards from a biological standpoint [16]. The past few years have been enlightening for the medical field regarding the effects that alcohol has on cardiovascular health. Viewpoints have been changed in the scientific community, biases from previous studies have been identified, and new observations have been made. This narrative review accentuates new results, the studies that led to them, and the potential biases that can still be encountered when it comes to this topic.

## 2. Materials and Methods

To offer a thorough synthesis of the current research on this subject, this narrative review seeks to address the current controversy surrounding the association between alcohol consumption and CVD. This literature review employs an interpretative narrative review methodology and draws from a comprehensive database of scientific materials. We prioritize an extensive assessment of the relevant research over comprehensive quantitative analyses. We used PubMed, Scopus, and Web of Science as our main sources of scientific literature. To narrow down the results, we used a combination of specific keywords and their synonyms, along with Boolean operators: (“alcohol consumption” OR “moderate drinking” OR “heavy drinking”) AND “cardiovascular disease” AND (“hypertension” OR “arrhythmia” OR “stroke” OR “cardiomyopathy”). Our synthesis highlights older results, key trends, ongoing debates, and shifts in medical understanding, providing insight into the changing perspectives on alcohol consumption and cardiovascular health (Table 1).

In addition to the interpretative narrative approach, this review also includes a descriptive analysis of the study characteristics in the mentioned studies, particularly concerning genetic risk factors, gender differences, and shifts in paradigms between earlier and more recent studies contributing to CVD. Studies were selected based on specific criteria, including studies that analyzed genetic predispositions related to CVD risk, studies that provided data from diverse geographic regions or countries to enable cross-country comparisons, and studies that explored the interaction between genetic factors and lifestyle elements such as alcohol consumption. We excluded studies that did not differentiate genetic variations or did not provide adequate geographical or population data. The selection of studies for inclusion in this review was conducted by three independent members of our research team. Each member screened the identified studies based on the predefined inclusion criteria. Only studies that were selected by all three reviewers were included in the final analysis to ensure objectivity and minimize bias in the selection process.

## 3. Molecular Mechanisms of Ethanol Metabolism and Their Impact on Cardiovascular Health

**Figure 1:**
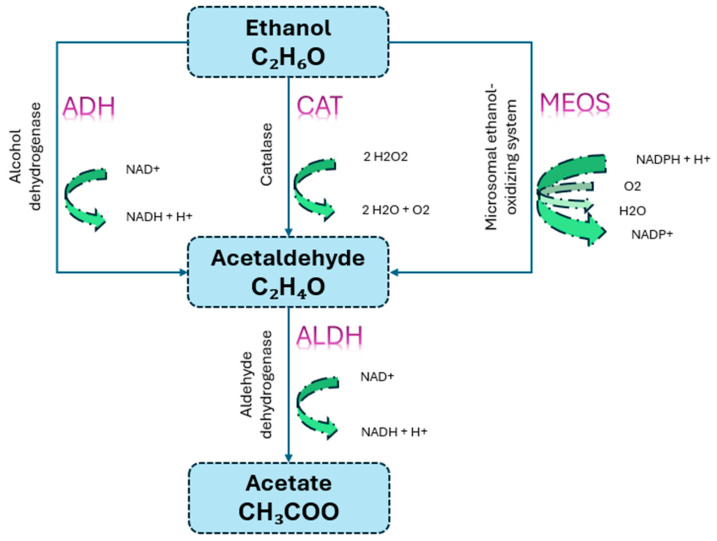
Ethanol metabolism pathways.

To fully understand the impact of alcohol use on cardiovascular health, it is important to comprehend the molecular processes involved in its metabolism. There are several biochemical pathways involved in the hepatic metabolism of ethanol which are responsible for both immediate effects and long-term ones that alcohol may have on cardiac tissues. Clarifying these processes allows for a deeper comprehension of the impact of alcohol on different facets of cardiovascular function, including lipid metabolism, endothelial function, and blood pressure regulation. The aforementioned observations explain the potential role of differences in metabolic pathways in the pathogenesis of alcohol-related cardiovascular diseases such as hypertension, cardiomyopathy, and arrhythmias [17]. Ethanol metabolization is a two-step process that takes place in the liver, involving acetaldehyde and acetate. Three metabolic pathways are involved: alcohol dehydrogenase (ADH), catalase (CAT), and the microsomal ethanol oxidizing system (MEOS). Alcohol dehydrogenases and aldehyde dehydrogenases catalyze the initial and subsequent stages of ethanol oxidation (Figure 1) [18,19,20]. Alcohol dehydrogenase is strongly attracted to ethanol but has a limited ability to bind to it. In individuals who consume large amounts of alcohol, the CAT pathway becomes more significant. MEOS, on the other hand, is increased with prolonged alcohol exposure [21]. Within the brain, ethanol undergoes oxidation facilitated by the CAT pathway [22]. As a result of chronic alcohol exposure, the rate of ethanol metabolism may increase, which is the cause of alcohol dependence. A faster elimination of ethanol can allow individuals to consume greater amounts of alcohol to reach the intended euphoric effects [23].

Acetaldehyde is not the only reactive result of ethanol metabolism. Cytochrome P450 enzymes produce reactive oxygen species (ROS). Antioxidants eliminate ROS, while glutathione S-transferases regulate secondary products. During detoxification, ADH and ALDH activity affect ethanol and acetaldehyde levels, generating oxidative stress and affecting cellular oxidative balance [24,25]. ALDH2 is a very important gene in the metabolism of acetaldehyde, a byproduct of alcohol, converting it into non-toxic acetic acid [26]. In humans, it can be found in three genotypes: wild-type, heterozygous, and variant-type homozygous [27]. One of these genotypes, the variant-type homozygous, can suffer a mutation that causes the ALDH2*2 gene which is prevalent in East Asians, making up 8% of the global population [28,29]. Those having the mutation have lower enzyme activity, affecting acetaldehyde detoxification. The clinical aspects related to the ALDH2*2 gene are face flushing and a higher heart rate, with Taiwanese men with the heterozygous ALDH2*1/*2 genotype being particularly sensitive to alcohol, as evidenced by their increased heart rate and face capillary blood flow [27,30].

A thorough understanding of these mechanisms is required for identifying possible treatment targets and developing effective techniques to reduce the negative cardiovascular effects of alcohol use. However, the association between alcohol use and cardiovascular health is still debated. Some studies suggest that there is no safe level of alcohol for cardiovascular health, while others have indicated potential cardiovascular benefits associated with moderate alcohol consumption, such as increased high-density lipoprotein cholesterol (HDL-C) levels and antioxidant benefits [31,32,33]. Previous studies have shown that moderate alcohol consumption can have cardioprotective effects via raising HDL-C and heme oxygenase-1 (HO-1) levels; however, HO-1 knockout mice did not demonstrate these beneficial effects [33].

The possible advantages of moderate alcohol use for people with the ALDH2*1/*2 genotype remain debatable despite these findings. People with this genotype may underreport their alcohol consumption and drinking habits, according to epidemiological research, which could add bias to the evaluation of alcohol’s effects. Consequently, to provide appropriate recommendations about the drinking habits of individuals with the ALDH2*1/*2 genotype, a more thorough understanding of the effects of moderate alcohol use on cardiovascular health and the underlying mechanisms is necessary [34,35].

## 4. Alcohol Consumption: Patterns and Definitions

Understanding the impact of alcohol consumption on cardiovascular health requires clear definitions of drinking patterns, as you can see in Table 2 [36]. According to the Current Guidelines of the National Institute on Alcohol Abuse and Alcoholism (NIAAA), moderate drinking is defined as women taking a maximum of one drink per day and males ingesting a maximum of two drinks per day [37]. A moderated level of consumption has been associated with improved lipid profiles and reduced risk of coronary artery disease [36]. Heavy drinking, on the other hand, is characterized by an intake of a minimum of three drinks per day or a minimum of seven drinks per week for women, while the numbers for men are a minimum of four drinks per day and a minimum of fourteen drinks per week [38]. This pattern of consumption has been linked to hypertension, cardiomyopathy, and increased mortality [17]. Consuming a large quantity of alcohol in a short period, typically more than five drinks within about two hours for men and more than four drinks for women is called binge drinking, and such episodes can lead to acute and chronic health issues, such as arrhythmias, stroke, and sudden cardiac death [39]. These definitions help to categorize and assess the varying effects of alcohol consumption on cardiovascular health, facilitating more targeted and effective public health interventions and clinical guidelines [15].

Alcohol consumption patterns vary significantly across different regions and cultures, influenced by socioeconomic factors, cultural norms, and government policies (Table 3) [40]. In high-income countries, alcohol consumption tends to be higher, with a significant portion of the population engaging in regular drinking, often within social settings [41]. These regions also report higher rates of binge and heavy drinking, particularly among young adults and middle-aged populations [42]. In contrast, low- and middle-income countries often exhibit more diverse patterns of alcohol consumption [43]. Cultural or religious beliefs may lead to lower overall consumption levels in some areas [44]. However, these regions may also experience rising alcohol use due to increasing economic development and globalization, which bring greater availability and social acceptance of alcohol [45]. In parts of Europe and the Americas, alcohol is frequently consumed as part of daily life, often integrated into meals and social gatherings. Mediterranean countries, for instance, are known for their moderate drinking habits, particularly wine consumption [46]. Meanwhile, Northern and Eastern European countries report higher rates of heavy and binge drinking, with spirits being the preferred choice [47]. Asia presents a varied landscape, with some countries like China and India showing increasing alcohol consumption trends due to economic growth and changing lifestyles [48]. In contrast, countries with strong religious or cultural prohibitions, such as predominantly Muslim nations, report lower alcohol consumption rates [49]. Africa displays a wide range of drinking behaviors, from high levels of abstinence in some regions to significant alcohol use in others, often associated with locally produced beverages. The patterns of consumption are also shaped by regulatory frameworks, public health policies, and the presence of informal alcohol markets [50]. Understanding these global patterns is crucial for tailoring public health strategies and interventions to address the specific challenges and risks associated with alcohol consumption in different cultural and socioeconomic contexts.

A complex interaction of factors, including individual traits, social and cultural norms, economic situations, and governmental regulations, influences drinking habits, which are significantly influenced by individual characteristics, such as age, gender, genetics, and psychological condition. In comparison to women, younger individuals and men tend to drink more heavily and binge more frequently [11]. Genetic predispositions can influence an individual’s alcohol metabolism and potential for alcohol dependency [51,52]. There may be a correlation between mental health issues, such as anxiety, stress, and depression, and increased alcohol intake as a coping strategy [53]. Drinking habits are strongly influenced by social and cultural standards, including societal conventions and cultural background. While drinking may be discouraged or outright forbidden in certain cultures, alcohol is a customary component of social events and celebrations in others. How and when people choose to drink is also influenced by peer pressure, societal acceptance, and family attitudes toward alcohol [40]. Access to alcohol and drinking habits are influenced by socioeconomic level and economic stability. In general, higher income levels are associated with higher alcohol use. On the other hand, financial difficulty and unemployment might cause people to either drink less alcohol because of their limited resources or drink more because they use it as a stress reliever [42]. The amount of consumed alcohol is heavily impacted by government laws, such as taxation, the minimum legal drinking age, and availability limits. Higher taxes and prices lower overall consumption and discourage binge drinking. Laws governing the advertising and sale of alcoholic drinks also influence the opinions and behaviors of the general population [54]. Drinking habits are influenced by the availability of alcohol in the surrounding area, including the quantity and density of pubs, restaurants, and liquor stores. Communities with more alcohol establishments typically have higher rates of alcohol drinking and related health issues [55]. Education and public awareness campaigns regarding risks and repercussions of alcohol use can alter people’s drinking habits. Higher-educated people are frequently more conscious of the negative effects of binge drinking on one’s health and may adopt more restraint in their drinking habits [56]. Drinking patterns are significantly influenced by social networks and interactions, including peer groups and family dynamics. Social support networks have the power to either reduce or increase drinking, depending on the attitudes that are prevalent within them [57]. Public health programs can be more effectively designed to target and mitigate dangerous drinking behaviors, encouraging healthier lives and lowering the burden of alcohol-related illnesses, by taking these aspects into account (Table 4).

## 5. Mechanisms of Alcohol’s Effects on the Cardiovascular System

The cardioprotective effects of alcohol are thought to be mediated through several complex mechanisms, involving not only intermediate cardiovascular endpoints like lipid levels, hypertension, and diabetes but also other pathways by which moderate alcohol consumption may reduce CVD risk.

### 5.1. Alcohol Consumption and CVD Incidents

It is well known that high-density lipoprotein cholesterol (HDL-C) provides significant protection against CVD and that alcohol consumption increases plasma HDL-C. A thorough meta-analysis of 47 randomized controlled trials (RCTs) revealed a dose–response relationship between plasma HDL-C levels and alcohol use, including high consumption (above 60 g/day) [58]. Serum HDL-C increased by 0.072 mmol/L for alcohol intake between 12.5 and 29.9 g/day, 0.103 mmol/L for alcohol intake between 30 and 60 g/day, and 0.141 mmol/L for alcohol intake over 60 g/day [58]. By affecting HDL functions, HDL-related metabolic variables, and HDL composition, alcohol may raise plasma HDL levels. For example, prolonged alcohol use can raise the concentrations of important HDL apolipoproteins, such as apolipoprotein A-1 and apolipoprotein A-2, as well as the quantity and percentage of HDL particles [59,60]. Alcohol may improve HDL’s atheroprotective properties, even if the exact processes by which moderate alcohol use reduces CVD risk by raising HDL are not entirely understood. HDL is in charge of reverse cholesterol transport, which is the mechanism by which peripheral free cholesterol is transported to the liver for elimination. By increasing HDL’s ability to absorb cholesterol, esterify cholesterol, and transport cholesteryl esters to the liver, alcohol intake may enhance HDL’s antiatherosclerotic effects [60]. Nonetheless, despite the rise in HDL levels, frequent alcohol use does not appear to have any further cardiovascular benefits. High blood pressure (BP) as a side effect of alcohol consumption may be responsible for the negative effects of alcohol on CVD [61].

As is often recognized, low-density lipoprotein cholesterol (LDL-C) poses a risk for atherosclerotic cardiovascular disease. There is no evidence of a correlation between alcohol intake and LDL-C levels in recent short-term RCTs [59]. Nevertheless, this correlation appears to change according to the population undergoing investigation. For instance, studies on Danish and Japanese populations showed a correlation between alcohol use and decreased blood LDL-C values [62,63]. On the other hand, research on older Turkish and Italian males revealed a correlation between elevated LDL-C levels and greater alcohol intake [64,65]. The lack of consistency in these results might be attributed to genetic factors particular to each allele [60,66]. For example, research conducted in Spain discovered that the link between increased LDL-C levels and alcohol use was limited to people who carried at least one copy of the Apo E4 allele. Similarly, a study from China discovered three genetic variants of the ApoA5 gene that may alter the link between alcohol consumption and LDL-C levels [64,67]. Additional investigations are required to comprehensively understand these intricate relationships.

While a number of experimental investigations have demonstrated that alcohol use in the short term can lower blood pressure, long-term alcohol consumption—more than one drink per day—might increase the chance of developing hypertension [68]. According to a meta-analysis of 12 studies, the relationship between alcohol use and the risk of hypertension varies between adult males and females [69]. In men, the risk of hypertension rises linearly with alcohol consumption, whereas in women, the relationship is more J-shaped. The meta-analysis found a linear dose–response relationship between alcohol use and hypertension risk in men, with RRs of 1.09 for 10 g/day of pure alcohol, 1.57 for 50 g/day, and 2.49 for 100 g/day. In contrast, women’s risk of hypertension was lower only at minimal alcohol intake (less than 10 g/day) but increased at moderate to high levels of consumption, with RRs of 1.10, 1.81, and 2.81 for 10 g/day, 50 g/day, and 100 g/day of alcohol, respectively [69]. Further RCTs have shown that alcohol has a negative effect on blood pressure. For example, Zilkens et al. discovered that drinking alcohol every day for four weeks increased ambulatory systolic blood pressure regardless of the type of alcoholic beverage. Specifically, daily use of beer (41 g of alcohol) and red wine (39 g of alcohol) increased 24 h systolic blood pressure by 1.7 mm Hg and 2.2 mm Hg, respectively, compared to four weeks of abstinence. The exact mechanism by which alcohol elevates blood pressure remains unclear [70]. One randomized controlled trial found that higher systolic blood pressure was associated with an elevated augmentation index (AI%), an important marker for central artery stiffness and an independent risk factor for hypertension. This shows that continuous alcohol use may contribute to elevated blood pressure by increasing vascular stiffness [71].

**Figure 2:**
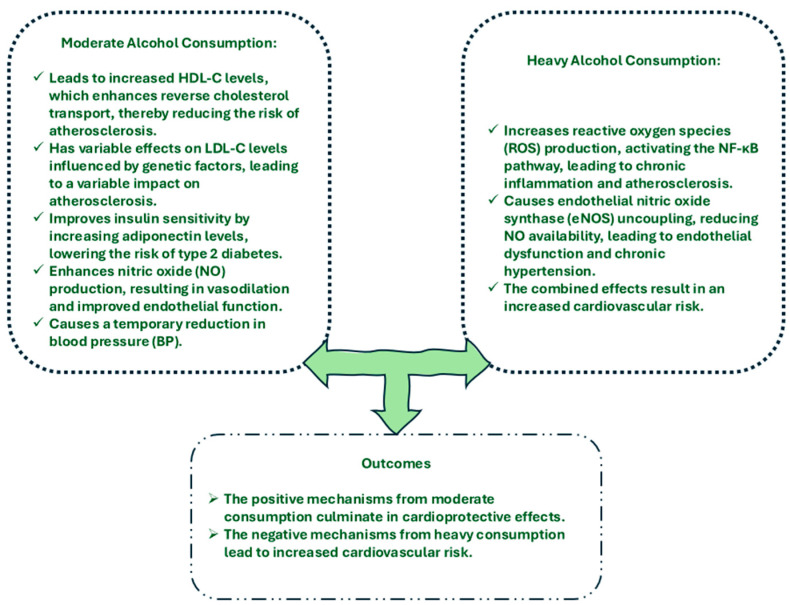
Mechanisms of alcohol consumption on cardiovascular system.

Based on older scientific studies and meta-analyses, moderate alcohol intake appears to have a beneficial effect on glucose metabolism and may lower the risk of developing type 2 diabetes (Figure 2) [72,73,74,75,76,77]. However, according to newer meta-analyses, the relationship between alcohol intake and diabetes risk is U-shaped, suggesting that excessive alcohol consumption may increase the risk of type 2 diabetes. The risk of acquiring type 2 diabetes in people who consumed less than 6 g of alcohol per day was 0.87 when compared to non-drinkers. Regarding moderate alcohol use, the relative risks were 0.70, 0.69, and 0.72 for 6 to 12 g/day, 12 to 24 g/day, and 24 to 48 g/day, respectively. Nevertheless, with an RR of 1.04, heavy drinkers who consume more than 48 g/day had a risk of DM comparable to non-drinkers [77]. Moderate alcohol consumption has been shown to have a positive impact on glucose metabolism and insulin sensitivity. This benefit is observed in both individuals with diabetes and those without it. One possible mechanism for this improvement is the role of adiponectin, a protein hormone that regulates glucose absorption and fatty acid oxidation [72,74,78]. Adiponectin levels are positively influenced by moderate alcohol use, according to multiple studies [79,80,81].

### 5.2. Molecular Mechanisms of Alcohol’s Effects on the Cardiovascular System

Alcohol use, particularly excessive amounts, can cause oxidative stress (OS) by increasing reactive oxygen species (ROS) production inside cells [82]. ROS can cause direct damage to proteins, lipids, and DNA in cardiovascular tissues [83]. Activation of the nuclear factor-kappa B (NF-κB) pathway is one of this OS’s main effects, and it has the potential to become a significant regulator of inflammation [84]. NF-κB activation increases pro-inflammatory cytokines such as TNF-α, IL-6, and CRP, which can lead to a chronic inflammatory state in blood vessels. Atherosclerosis develops because of the endothelium lining being harmed by this inflammation. Plaque formation is accelerated over time by the ongoing oxidative and inflammatory environment, increasing the risk of heart attacks and strokes [85].

The endothelium produces nitric oxide (NO), a signaling molecule that has been proven to assist in blood vessel dilatation and consequently inhibit platelet aggregation, which is essential for preserving vascular health [86]. Endothelial function is impacted by alcohol in a dose-dependent manner. By altering the levels of endothelial nitric oxide synthase (eNOS), moderate alcohol consumption can increase the production of NO and potentially protect the cardiovascular system. Chronic heavy drinking, on the other hand, has the opposite impact. When eNOS is uncoupled by too much alcohol, the enzyme produces more ROS, especially superoxide, rather than NO [87]. This further impairs endothelial function by increasing oxidative stress in addition to lowering NO availability. Endothelial dysfunction, as a result, raises the risk of cardiovascular events by increasing vascular resistance, hypertension, and the tendency for clot formation [88].

## 6. Alcohol’s Effects on Cardiovascular Health

### 6.1. Ischaemic Heart Disease

The high prevalence of alcohol use and ischemic heart disease (IHD) in high-income countries has generated considerable research interest. Multiple systematic reviews, meta-analyses, and individual studies have examined the influence of alcohol use on ischemic heart disease (IHD), specifically focusing on myocardial infarction, which is the most prevalent, frequent, and fatal subtype of IHD [61,66,89,90,91,92,93,94].

For a long time, it was believed that moderate alcohol use could help prevent IHD. Early research suggested that alcohol—especially beer—was linked to a lower incidence of myocardial infarction (MI) and other CHD-related outcomes. Studies from Sweden (men who consumed alcohol had a 40% lower incidence of MI than non-drinkers) and Germany (which showed an HR of 0.51 for drinkers in comparison with non-drinkers) supported this theory [95,96]. Research conducted in the United States revealed that male physicians who moderately drank had a lower incidence of MI and angina pectoris [97]. Larger assessments, such as the NHANES I epidemiologic follow-up research, suggested a J-shaped curve, showing moderate drinkers to have a reduced risk of CHD than non-drinkers, further corroborating this [98,99]. A prospective study of male British GPs found that drinkers have a lower risk of all-cause death and coronary heart disease (CHD) mortality compared to non-drinkers while a meta-analysis of 44 observational studies found a J-shaped association between alcohol use and CHD risk. The lowest risk was found at one to two drinks per day, with the lowest risk observed at one to two drinks per day [100,101]. Research involving middle-aged males from France and Northern Ireland revealed that, as compared with non-drinkers, French individuals in the highest quartile of alcohol intake had a substantially reduced risk of angina pectoris, MI, and total CHD events. On the other hand, in Northern Ireland, alcohol’s protective effect was exclusively noted for MI [102].

Recent research has led to a reevaluation of previous conclusions as the scientific community further investigated the effects of alcohol on cardiovascular health. New research has cast doubt on the extent to which alcohol protects against coronary heart disease (CHD), painting a more nuanced picture than was previously believed. Not all research, has found a J- or U-shaped correlation between alcohol and CHD. An increasing database of recent research indicates that the stated benefits of moderate alcohol intake may not be as significant as previously thought. Ding et al. discovered that limiting alcohol consumption to around 105 g per week was related to lower rates of death and cardiovascular morbidity. This threshold is significantly lower than the amounts recommended by most contemporary standards [103]. Similarly, Holmes et al. used Mendelian randomization to investigate the causal relationship between alcohol and cardiovascular outcomes and discovered that people with a genetic variant linked to lower alcohol consumption or abstinence had a better cardiovascular profile and a lower risk of coronary heart disease than those without the variant. These data suggest that even among light to moderate drinkers, lowering alcohol intake can be beneficial for cardiovascular health [104]. Russia’s statistics consistently point to a negative link with the outcomes of CHD, which may be explained by the prevalent drinking habits of the country, which are more likely to involve frequent heavy drinking than routine, moderate intake [105,106].

Recent research has also revealed the dangers associated with various drinking patterns. For example, while moderate drinking was originally thought to provide cardiovascular advantages, excessive episodic drinking—defined as consuming a high amount of alcohol in a short period—has been linked to increased cardiovascular risk [107,108]. Any potential protective benefits of moderate consumption can be negated by this drinking pattern, which may also raise the risk of ischemic accidents overall.

Significant obstacles in alcohol-related studies have included unmeasured confounding factors and biases. The J-shaped curve frequently seen in studies has been explained by the “sick-quitter” theory, which postulates that former drinkers may exaggerate the hazards associated to lifetime abstainers [109]. Epidemiological studies have shown that individuals who abstain from alcohol for their whole lives do not have a significant correlation with IHD morbidity. However, those who have previously consumed alcohol are more likely to experience IHD mortality [110].

More caution and refinement are now applied to our knowledge of alcohol’s relationship to CHD than in the past. Newer studies emphasize the significance of taking potential dangers and biases into account, even if prior studies supported the notion that moderate alcohol use could lower the risk of CHD. The J-shaped curve, which shows a decreased risk of cardiovascular disease at moderate alcohol intake, is still significant, although the total advantages might not be as great as previously believed. Furthermore, the negative consequences of severe episodic drinking and the impact of confounding variables underscore the necessity of a more sophisticated strategy regarding alcohol intake and cardiovascular health. It is critical to take a balanced view of alcohol intake as science advances. Even though there may still be some cardiovascular benefits to moderate drinking, it is important to understand the hazards involved and consider each person’s unique health history and drinking habits. This evolving knowledge highlights the importance of ongoing studies and a responsible approach to alcohol use concerning heart health.

### 6.2. Hypertension

Numerous studies have examined the link between alcohol intake and hypertension, a major public health issue [111]. Multiple meta-analyses and research have examined this link over the past few decades, revealing deeper findings with larger and more diverse datasets [69,112,113,114,115,116,117].

Older studies showed that very low alcohol use in women reduced hypertension risk modestly, though still statistically relevant. Some studies found that women who drank one drink per day had a slightly decreased incidence of hypertension than abstainers. Men did not benefit from this protection [117].

Recent and extensive meta-analyses broadened this view. Alcohol use enhanced men’s hypertension risk at any level, according to a meta-analysis. Women who consumed up to 24 g of alcohol per day were not in danger, but more alcohol intake raised the risk. Former drinkers had the same risk as lifelong abstainers. Men with 60 g of alcohol per day had a considerably greater risk of hypertension. The difference in risk for alcohol consumption up to 24 g per day between men and women may indicate more hazardous drinking when it comes to men. The alcohol–hypertension link has become better understood after more research [118,119]. A 2023 meta-analysis examined how alcohol affects blood pressure across demographics. It demonstrated that even moderate alcohol drinking (1–2 drinks per day) increased hypertension risk in men and women, contradicting earlier studies that suggested a protective benefit at low consumption levels [120]. The risk was dose-dependent, with higher intake levels increasing blood pressure [121]. Alcohol affects blood pressure through mechanisms that have been studied. Alcohol consumption activates the sympathetic nervous system, causing vasoconstriction and cardiac output, which raise blood pressure [122]. Alcohol also disrupts RAAS control, increasing hypertension risk [123].

Alcohol use is known to cause hypertension. Alcohol reduction consistently lowers systolic and diastolic blood pressure dose-dependently in experimental investigations. Reducing alcohol consumption by 50% in heavy drinkers (72 g/day) resulted in a mean systolic blood pressure drop of −5.50 mmHg. This clinically substantial reduction shows that alcohol reduction may be an important hypertension therapy strategy, especially for heavy drinkers [119,124].

These results have major public health implications. Although minimal alcohol use was thought to protect against hypertension, recent research suggests that even moderate drinking may raise the risk, particularly in men. These findings state the importance of alcohol reduction as a hypertension prevention approach and suggest that public health guidelines should emphasize the dangers of moderate alcohol use. Reducing alcohol consumption, especially among heavy and binge drinkers, could significantly lower hypertension prevalence and improve cardiovascular outcomes [124,125].

### 6.3. Stroke

The relationship between alcohol consumption and stroke has been the subject of extensive research. Stroke can be broadly categorized into two primary subtypes, each with distinct etiologies—ischemic stroke (IS), which arises from ischemic processes, and hemorrhagic stroke (HS), which results from bleeding events—each of them with different medical approaches and treatments [126]. Due to its higher prevalence, IS often dominates research on overall stroke incidence [127]. Given the etiological similarities between IS and ischemic heart disease (IHD), one might anticipate a comparable relationship between alcohol consumption and IS [128]. Evidence indicates that moderate alcohol intake may offer protection against ischemic stroke, though not against hemorrhagic stroke [129].

Based on multiple meta-analyses, both early studies and more recent ones, there is a J-shaped relationship between alcohol consumption and the risk of ischemic stroke [113,130,131,132]. This means that light to moderate alcohol consumption is associated with a lower risk of ischemic stroke compared to abstaining from alcohol. However, heavy drinking significantly increases the risk of stroke. Excessive alcohol consumption is also linked to a higher risk of hemorrhagic stroke [133]. A meta-analysis of 16 case–control and 19 prospective studies revealed that consuming up to 12 g of alcohol per day (approximately one drink) was associated with a 17% reduction in stroke risk. In contrast, consuming more than 60 g per day (equivalent to 4–5 drinks) was linked to a 64% increase in stroke risk compared to non-drinkers [134]. A Japanese study that studied the relationship between alcohol intake and myocardial infarction also found no significant differences in ischemic stroke risk across alcohol levels [135].

Regarding subarachnoid hemorrhage (SAH), one meta-analysis reported that light to moderate alcohol consumption (less than 150 g per week) did not affect SAH risk. In both longitudinal and case–control studies, heavy drinking was associated with a significantly elevated risk of SAH [136]. Berger et al. found that light to moderate alcohol consumption was responsible for a reduction of approximately 20% in the overall risk of stroke, including ischemic stroke, in a cohort of male physicians [137]. Sacco et al. who conducted a multiethnic case–control study, identified an independent, inverse association between moderate alcohol consumption and IS, while observing that individuals who consumed several drinks per day faced a heightened risk of IS [138].

Based on a meta-analysis of 27 prospective cohort studies, it was found that moderate alcohol consumption, up to 24 g per day, is associated with a reduced risk of IS compared to abstaining from alcohol. However, the risk of IS increases with alcohol consumption above 24 g per day [59]. Considering the research results, it can be concluded that the likelihood of experiencing hemorrhagic strokes, particularly intracerebral and subarachnoid hemorrhages, rises with each further drink consumed. Exceeding a daily intake of 48 g greatly increases the likelihood of experiencing these specific kinds of strokes [131,139].

Moreover, several studies have highlighted that heavy episodic drinking significantly increases the risk of both IS and HS [140,141,142], with some evidence suggesting that the risk escalates with the frequency of such drinking episodes [141]. Alcohol consumption is also recognized as a precipitating factor for stroke events, with higher intake within 24 h or over a week correlating with an increased risk of both IS and HS [143].

### 6.4. Atrial Fibrillation and Cardiomyopathy

Alcoholic cardiomyopathy is defined as a form of dilated cardiomyopathy resulting from prolonged heavy alcohol consumption and is acknowledged as a distinct clinical condition by the World Health Organization, with its own International Classification of Diseases, Tenth Revision (ICD-10) code (I42.6). Despite this classification, it remains a diagnosis of exclusion. Clinically and histologically, alcoholic cardiomyopathy is indistinguishable from idiopathic dilated cardiomyopathy, making a thorough patient history crucial for diagnosis. The specific dose of alcohol necessary to induce cardiomyopathy is not well established in epidemiological studies, though review articles frequently cite a daily intake of 80 to 90 g (approximately eight drinks) over five years as a common threshold [144,145].

This estimate is derived from extrapolations in selected case–control studies, where patients hospitalized with cardiomyopathy were interviewed regarding their alcohol consumption [146]. A 2017 systematic review aimed to quantify the relationship between alcohol intake and the risk of developing cardiomyopathy but concluded that the available data were insufficient for a meaningful analysis. Some smaller clinical studies have suggested that women may develop alcoholic cardiomyopathy at lower levels of alcohol consumption compared to men, although the precise differences in the alcohol dose required to cause the disease remain unknown [147,148].

Based on the given evidence, it can be stated that there exists a correlation between the intake of alcohol and the susceptibility to atrial fibrillation (AF) [149,150,151,152]. Extensive meta-analyses and cohort studies have consistently demonstrated that even modest alcohol intake is linked to a higher likelihood of atrial fibrillation (AF). Nevertheless, there is some inconsistency in the results, since one study indicates that there is no notable rise in arterial fibrillation (AF) risk for moderate alcohol intake [149]. Moreover, a J-shaped correlation has been identified, suggesting that individuals who consume a moderate quantity of alcohol, particularly wine, had the lowest odds of developing atrial fibrillation (AF). In addition, decreasing alcohol use has been associated with a decreased recurrence rate of atrial fibrillation [153,154].

Alcohol consumption, whether regular or sporadic, can contribute to hypertension, structural damage to the heart muscle, and arrhythmias. It can induce cardiomyopathy, characterized by ventricular dilation, hypertrophy, and functional impairment [155]. Consumption exceeding 80 g per day significantly raises the risk. Lower daily alcohol consumption and shorter duration of intake over several years can have similar adverse effects in women as in men. Estimates suggest that a significant percentage of individuals with alcohol use disorder may develop cardiomyopathy and a significant percentage of those with dilated cardiomyopathy may have alcoholic cardiomyopathy [156].

### 6.5. Heart Failure

Cardiovascular disease categories, including IHD, hypertension, and cardiomyopathy, are known to elevate the risk of heart failure. Several meta-analyses have consistently addressed this issue [157,158,159]. A recent meta-analysis, published in 2018, analyzed data from 355,804 participants and identified 13,738 cases of heart failure across 13 cohort studies. This analysis revealed a curvilinear dose–response relationship between alcohol consumption and heart failure risk [158]. Specifically, compared to non-drinkers, the RR for different levels of weekly alcohol consumption were as follows: 1–84 g (RR = 0.86), 85–168 g (RR = 0.88), 169–336 g (RR = 0.91), and greater than 336 g (RR = 1.16). Based on the meta-analysis, it can be concluded that former drinkers have a higher risk of heart failure compared to lifetime abstainers. However, the influence of sex and other potential factors on this risk is still uncertain due to limitations in the available data [158].

The American Heart Association’s scientific statement on specific dilated cardiomyopathies identifies alcohol as one of the primary contributors to the development of cardiomyopathy [160]. Similarly, the 2021 guidelines from the American Heart Association (AHA) and the American College of Cardiology (ACC) recognize alcohol as a significant risk factor for heart failure [161]. Although extensive evidence links excessive alcohol consumption to subclinical cardiac remodeling and impaired diastolic function, as observed through echocardiographic assessments, longitudinal community-based studies have often reported a seemingly paradoxical reduction in heart failure risk associated with moderate alcohol intake, and no significant risk increase with heavy alcohol use compared to abstinence [162,163,164,165,166,167]. Conversely, conditions with a well-established association with alcohol consumption, such as liver cirrhosis and atrial fibrillation, exhibit a more direct correlation with alcohol intake, where even one daily drink elevates the risk [151,168,169,170].

The biological mechanisms behind this paradoxical U-shaped or inverse relationship between alcohol consumption and heart failure are challenging to reconcile, particularly if alcohol is indeed a common cause of cardiomyopathy and heart failure in the general population. A recent Mendelian randomization study further supports this complexity, finding no association between genetically predicted alcohol consumption and increased heart failure risk, despite established links to higher risks of stroke and atrial fibrillation [171]. This evidence suggests that alcohol may not be as frequent a cause of cardiomyopathy and heart failure as previously thought. Moreover, research has indicated that a significant proportion of individuals diagnosed with alcohol-induced cardiomyopathy may possess genetic variants associated with dilated cardiomyopathy, potentially indicating misclassification or that alcohol exacerbates cardiomyopathy in genetically predisposed individuals [172,173].

It is also plausible that earlier population-based studies were confounded by socioeconomic and lifestyle differences between drinkers and non-drinkers, although Mendelian randomization studies are less vulnerable to such confounding factors. A more recent investigation utilizing individual-level data from 19 high-income countries, focusing exclusively on individuals who consume alcohol, found that consuming more than 100 g of alcohol per week was associated with an increased risk of heart failure, with no protective effect observed for moderate alcohol consumption compared to light use [31]. Additionally, an analysis of hospitalization data from the Healthcare Cost and Utilization Project database revealed that patients with documented alcohol abuse had a twofold increased relative risk of heart failure compared to those without alcohol abuse [174].

### 6.6. Peripheral Arterial Disease

Two studies have demonstrated an inverse relationship between moderate alcohol consumption and the risk of peripheral arterial disease (PAD). Djousse et al. found that the RRs of developing intermittent claudication, a symptom of PAD, were significantly lower in moderate drinkers compared to non-drinkers, with RRs of 0.67 for men and 0.44 for women [175]. Similarly, Camargo et al. reported that moderate alcohol intake was associated with an approximately 25% reduction in PAD risk compared to abstinence from alcohol [176]. However, a separate study found no significant association between alcohol consumption and PAD among diabetic adults [177]. Additionally, research conducted in the Netherlands suggested that consuming 1-2 drinks per day might lower the risk of amputation resulting from PAD [178].

## 7. Summary and Future Directions

The field of cardiovascular health is constantly changing, and to improve public health initiatives and individualized interventions, as well as to gain a more comprehensive understanding of the long-term effects of alcohol consumption on cardiovascular health, there are several important research gaps and emerging areas that require further investigation. While a great deal of research has been conducted on the short- and immediate-term effects of alcohol, little is known about the long-term chronic effects of moderate and heavy drinking (Table 5). Studies that follow participants over an extended period are necessary to clarify how chronic alcohol use affects cardiovascular risk factors such as hypertension, atherosclerosis, and heart failure [179]. Understanding these long-term effects is fundamental for accurate risk assessment and the development of preventive measures.

Alcohol use is currently seen from three distinct perspectives: as a primary cause of CVD, as something whose effects may be reversed by abstinence, and as something that directly damages cardiac tissue. These viewpoints are crucial to the current discussions in the subject [180]. There are few large-scale case-control studies and no conclusive links between alcohol use and heart failure in more comprehensive, community-based research, despite smaller studies suggesting a high incidence of alcohol drinking in individuals with dilated cardiomyopathy [163,164]. Anecdotal data and limited cohort studies show improvements in left ventricular ejection fraction, supporting the idea that abstinence can repair alcoholic cardiomyopathy [181,182]. However, evidence of permanent fibrosis in cardiac biopsies, as well as comparable recovery patterns in idiopathic cardiomyopathy following medical therapy, suggests that abstinence alone may not be sufficient to reverse heart damage [17,183]. Furthermore, while both animal and human studies support the direct toxic impact of alcohol on myocardial tissue, counterarguments suggest that alcohol-induced hypertension and hepatic alterations may also contribute to changes in cardiac structure and function, indicating a more complex interplay of factors in the pathogenesis of alcoholic cardiomyopathy [168,170]. Such controversies draw attention to the necessity of conducting more extensive research to elucidate the processes and risk factors producing alcoholic cardiomyopathy.

**Table 5:** Future directions.

Aspect	Key Findings	Future Research Directions
Long-term Impact	Few studies have been conducted on the long-term consequences of moderate to heavy alcohol use on cardiovascular health.	Conduct longitudinal studies to track long-term effects on hypertension, atherosclerosis, and heart failure [179].
Role in Cardiovascular Disease	Alcohol is a major cause of cardiovascular disease with potential reversibility through abstinence.	Investigate large-scale case–control studies to clarify associations with heart failure and evaluate the effectiveness of abstinence in reversing alcoholic cardiomyopathy [180].
Toxic Effects on Myocardial Tissue	Evidence supports alcohol’s direct toxic impact on myocardial tissue; debated factors include hypertension and hepatic alterations.	Explore complex interactions between alcohol-induced hypertension, hepatic alterations, and myocardial damage [17].
Concurrent Substance Use	Research needed on how concurrent use of substances (e.g., cocaine and tobacco) affects cardiomyopathy risk.	Perform pharmacoepidemiological studies and clinical trials to assess combined effects on cardiovascular health [184].
Cancer Risks	Alcohol classified as a Group 1 carcinogen; even moderate consumption increases cancer risk.	Examine the impact of alcohol on cancer risk relative to cardiovascular benefits and reassess public health strategies [185].

Moreover, research should also focus on how the concurrent use of other substances, such as cocaine and tobacco, might affect the risk of developing cardiomyopathy in individuals with alcohol use disorder [184]. To determine whether conventional heart failure therapies are effective in delaying the onset and progression of alcoholic cardiomyopathy in at-risk populations, pharmacoepidemiological research and RCTs are also necessary. Until more conclusive evidence emerges, caution should be exercised in diagnosing alcoholic cardiomyopathy to ensure that other potential contributing factors, including genetic predispositions, are not overlooked. Addressing these unresolved questions and exploring new research avenues are crucial for advancing our understanding of alcohol’s impact on cardiovascular health and for developing targeted, evidence-based public health strategies and clinical practices. As the field of alcohol and cardiovascular health continues to evolve, there are significant gaps and emerging areas of research that require further exploration. Addressing these unresolved questions and exploring new research avenues is crucial for a more comprehensive understanding of the long-term cardiovascular effects of alcohol and for developing more effective public health strategies and personalized interventions.

On the other hand, recent research has increasingly shown a strong link between alcohol consumption and various types of cancers, challenging earlier perceptions of alcohol’s health impacts [186]. Alcohol is classified as a Group 1 carcinogen by the World Health Organization (WHO), which emphasizes that even moderate alcohol consumption can increase cancer risk [185]. This includes increased risks for cancers such as breast, liver, and those in the digestive system [187]. The WHO’s position highlights that even moderate drinking can raise cancer risk, which has shifted the focus away from any perceived cardiovascular benefits. This underscores the need to consider cancer risks seriously when evaluating the overall health effects of alcohol.

## 8. Conclusions

In conclusion, the relationship between alcohol consumption and cardiovascular health is multifaceted, with both potential benefits and significant risks. Moderate drinking—defined as one to two drinks per day—has been linked to a lower risk of ischemic heart disease and stroke, indicating some protective effects. However, these advantages are outweighed by the risks associated with heavy or episodic drinking, such as hemorrhagic stroke, atrial fibrillation, and cardiomyopathy.

The impact of alcohol varies based on factors like drinking patterns, age, gender, and individual health conditions, highlighting the need for a nuanced approach to its effects.

The observed J-shaped relationship between alcohol consumption and cardiovascular conditions underscores the complexity of these effects, pointing to the necessity for personalized guidance. Future research should focus on clarifying the dose–response relationship, exploring long-term effects, and investigating the underlying mechanisms at molecular levels. Additionally, refining public health guidelines to match individual risk profiles is crucial. Addressing these areas will enhance our understanding and lead to more effective strategies for managing alcohol-related cardiovascular risks.

## Figures and Tables

**Table 1:** Overview of study design and analysis.

Aspect	Details
Objective	To review the association between alcohol consumption, genetic predispositions, and CVD, considering alcohol quantity, gender differences, and shifts in findings over time.
Study Design	Narrative review with descriptive analysis of key study characteristics.Focus on qualitative synthesis.
Databases Used	PubMed, Scopus, Web of Science.
Search Keywords	Alcohol consumption, moderate drinking, heavy drinking, cardiovascular disease, hypertension, arrhythmia, stroke, cardiomyopathy.
Inclusion Criteria	Studies on genetic predispositions, alcohol quantity, regional comparisons, gender differences, and older vs. more recent studies.
Exclusion Criteria	Studies lacking data on genetic variation, geography, alcohol quantity, or gender-specific effects.
Study Selection Process	Three independent reviewers selected studies; only common selections were included to reduce bias.
Bias Minimization	Multiple reviewers, predefined inclusion criteria, and consensus on study inclusion ensured objectivity.
Data Analyzed	Genetic risk, regional variation, alcohol quantity, gender effects, and temporal shifts in research.
Descriptive Focus	Study design, population, geographic data, alcohol consumption levels, gender differences, and shifts in findings.
Analysis Approach	Narrative synthesis of trends, descriptive summary of study characteristics, and comparison of older vs. newer findings.
Data Presentation	Tables and charts summarizing study characteristics, genetic factors, alcohol consumption, and gender effects.
Conclusion	Insight into genetic, environmental, and lifestyle factors, with focus on alcohol quantity, gender, and evolving CVD perspectives.

**Table 2:** Alcohol consumption patterns and definitions.

Category	Definition	Impact on Cardiovascular Health
Moderate Drinking	No more than 1 drink/day for women; no more than 2 drinks/day for men	Associated with some cardioprotective effects (improved lipid profiles and reduced CAD risk) [37].
Heavy Drinking	More than 3 drinks/day or more than 7 drinks/week for women; more than 4 drinks/day or more than 14 drinks/week for men	Linked to negative cardiovascular outcomes (hypertension, cardiomyopathy, and increased mortality) [38].
Binge Drinking	More than 5 drinks within 2 h for men; more than 4 drinks within 2 h for women	Can lead to acute and chronic health issues (arrhythmias, stroke, and sudden cardiac death) [39].

**Table 3:** Global alcohol consumption patterns.

Region	Consumption Trends	Cultural and Economic Factors
North America	Higher regular drinking, often in social settings; increased binge and heavy drinking	Economic stability and social norms promoting regular drinking [41].
Europe	Moderate drinking in Mediterranean regions (wine with meals); higher binge drinking in Northern/Eastern Europe	Cultural integration of alcohol in daily life, with regional preferences for wine/spirits [46,47].
Asia	Rising consumption in countries like China and India; lower rates in regions with religious prohibitions	Economic growth, lifestyle changes, and cultural/religious restrictions in some areas [48].
Africa	Varied drinking behaviors: high abstinence in some regions and significant alcohol use in others	Locally produced beverages, cultural norms, and informal alcohol markets [50].
Oceania	Diverse consumption patterns: increasing alcohol use due to globalization	Cultural/religious influences and economic development increase alcohol availability [43].

**Table 4:** Factors influencing drinking behavior.

Factor	Details	Impact on Drinking Behavior
Individual Factors	Age, gender, genetics, mental health	Younger adults, males, and those with genetic predispositions more likely to drink heavily [11].
Social and Cultural Norms	Peer pressure, societal acceptance, cultural traditions	Drinking habits shaped by cultural background and societal attitudes toward alcohol [40].
Socioeconomic Status	Economic stability, disposable income, unemployment	Higher income leads to increased consumption; economic hardship can either reduce or increase drinking [42].
Government Policies	Taxation, legal drinking age, advertising restrictions	Higher taxes reduce consumption; regulations shape public perception and behavior [54].
Availability	Density of alcohol outlets in the community	More outlets lead to higher consumption rates and related health issues [55].
Public Awareness and Education	Health education campaigns and knowledge of alcohol risks	Higher education levels often correlate with more moderate drinking behaviors [56].
Social Networks	Family dynamics, peer groups, social support systems	Support systems can mitigate or exacerbate drinking behaviors [57].

## Data Availability

Not applicable.
